# Effects of Early Nutrition of Hatched Chicks on Welfare and Growth Performance: A Pilot Study

**DOI:** 10.3390/ani11102888

**Published:** 2021-10-03

**Authors:** Jan Berend Lingens, Amr Abd El-Wahab, Marwa Fawzy Elmetwaly Ahmed, Dana Carina Schubert, Christian Sürie, Christian Visscher

**Affiliations:** 1 Institute for Animal Nutrition, University of Veterinary Medicine Hannover, Foundation, Bischofsholer Damm 15, D-30173 Hannover, Germany; amrwahab5@mans.edu.eg (A.A.E.-W.); dana.carina.schubert@tiho-hannover.de (D.C.S.); christian.visscher@tiho-hannover.de (C.V.); 2 Department of Nutrition and Nutritional Deficiency Diseases, Faculty of Veterinary Medicine, Mansoura University, Mansoura 35516, Egypt; 3 Department of Hygiene and Zoonoses, Faculty of Veterinary Medicine, Mansoura University, Mansoura 35516, Egypt; marwafawzy@mans.edu.eg; 4 Farm for Education and Research Ruthe, University of Veterinary Medicine Hannover, Foundation, Schäferberg 1, D-31157 Sarstedt, Germany; christian.suerie@tiho-hannover.de

**Keywords:** chicks, early feeding, litter quality, foot pad health, growth

## Abstract

**Simple Summary:**

It is common practice that one-day-old chicks can be deprived of feed for about 48 h or more before they are housed on farms. Thus, we hypothesized that early nutrition on-farm hatched chicks might overcome the adverse effects of delayed nutrition on-hatchery hatched chicks regarding some animal welfare issues such as foot pad health as well as growth performance of birds. Our results confirmed that early nutrition on-farm hatched chicks together with using new fresh litter at d 7 of life led to a reduction in the severity of foot pad lesions and improved the growth performance of broiler chickens.

**Abstract:**

This study aimed to investigate the possibility of rearing newly hatched chicks with immediate access to feed and water in the same hatching unit one week prior to transferring them to the conventional broiler house with special regards to foot pad health and growth performance. Two trials were performed with a total of 6900/6850 (trials 1/2) broiler chickens (ROSS 308). A total of 3318/3391 chicks (trials 1/2) were transported from the hatchery (duration of about 3 h) and reared in a conventional broiler house (control group: delayed nutrition on-hatchery hatched). The control group did not receive any form of nutrition until they were taken to conventional broiler housing. Additionally, a total of 3582/3459 (trials 1/2) embryonated eggs (d 18) were obtained from the same parent flock of the same commercial hatchery and taken to the farm facility. After on-farm hatch, the chicks had immediate access to water and feed (experimental group: early nutrition on-farm hatched). After d 6/7 of life, the on-farm hatched chicks (trials 1/2) were transferred to the broiler house on the same facility. The delayed nutrition on-hatchery hatched groups displayed a significantly lower dry matter content in the litter compared to the early nutrition on-farm hatched groups (two-factorial analysis) at d 6/7 and d 14 of life. However, thereafter, no significant differences were noted. Based upon two-factorial analysis, the early nutrition on-farm hatched groups revealed lower foot pad lesions from d 14 of life onwards and showed a higher body weight (BW) throughout the rearing period compared to the delayed nutrition on-hatchery hatched groups (*p* < 0.05). Overall, early nutrition on-farm hatched chickens is of critical importance together with using new litter at d 7 to maintain healthy foot pads as well as to enhance nutrient utilization and optimize the growth performance.

## 1. Introduction

In commercial hatcheries, broiler chicks are usually hatched in conventional hatchers where they are not provided with feed and water until being housed on-farm (delayed nutrition) [1]. Eggs do not hatch at exactly the same time but do so within a “hatch window” which is defined as the time interval from the first to the last hatched chick [2]. This could be varied over a 24 to 48 h-period until the majority of chicks have been hatched [2]. This time may be further delayed depending on other procedures taking place within the hatchery such as sexing, vaccination, packaging and transportation distance to the farm [3]. During this period, the hatched chicks depend on residual yolk for maintenance and growth [4]. However, despite the presence of the yolk sac in chicks, prolonged post-hatch holding periods and delayed feeding may cause potential dehydration and energy depletion, which can have negative consequences on the growth and development of broiler chicks [5,6]. The time until the first feed and water intake “holding period” may take up to 72 h when long transportation distances are involved [7]. The long transportation process could exacerbate the depletion of reserves and dehydration through excessive thermoregulatory demands and stress, thus possibly affecting chicks’ BW and mortality rates [8,9]. The European Union legislation [10] specifies that newly hatched broiler chicks can be transported in the first 72 h after hatching including a maximum transport time of 24 h without feed and water. This recommendation is based on the fact that the chick’s metabolic reserves stored in the yolk sac last up to 3 d [11]. Nevertheless, chick mortality during the first week of life can reflect the stress of the transportation process [12]. Moreover, increasing mortality is considered to be the most extreme consequence of delayed feeding [3,13].

Delayed nutrition is only one of several welfare risks associated with conventional hatchery hatching. Exposure to continuous darkness in the hatcher, high noise and dust levels and transportation may act as stressors that impact chick welfare negatively [14,15,16]. Thus, any negative consequences resulting from delayed nutrition on the development of the intestine and muscles [17,18,19] and the immune system such as poor vaccination or reducing the response toward diseases [20] are considered welfare issues and should be avoided.

In recent years, early nutrition strategies, directly after hatching, have been proposed and conducted as an alternative to overcome the drawbacks of delayed nutrition on chick health and performance [21]. One strategy of early nutrition is giving the feed to the chicks in the hatchery, during transportation and before housing on-farm [22]. In the on-farm system, eggs are transported to the farm at d 18 of incubation, where the hatching period takes place on the farm and the hatched chicks have immediate access to feed and water directly after hatching and continuous light is provided [23]. Additionally, the on-farm system avoids the transport of newly hatched chicks from hatch chicks directly on-farm and hence decreases stress during handling [24]. All vaccinations (via drinking water or spray vaccinations) are performed on-farm [1]. Currently available on-farm hatching systems differ in the layout and degree of automation [25]. The use of these approaches is necessary not only for improving the growth performance but also for maintaining homeostasis [21,26]. Recent studies carried out in Belgium [24,25] assessed this system regarding BW, mortality and feed conversion ratio and found similarities between both on-farm hatched and commercially hatched flocks.

In addition, another welfare aspect, the birds’ foot pad health, was investigated by de Jong et al. [24,25]. Foot pad dermatitis (FPD) for example occurs if housing and management factors are disadvantageous [27]. There are many factors influencing the occurrence and severity of FPD. Nonetheless, poor litter quality, i.e., wet litter, is the most important cause of FPD [28,29,30]. Thus, achieving good litter quality and a low prevalence of FPD is highly desirable regarding the health and welfare of the animals.

It was hypothesized that a reduction in stressors in the peri- and post-hatching environments, such as the absence of deprivation of feed and water and less or no handling, would lead to improved welfare both early and later in life. Early nutrition on-farm hatched chicks were expected to score best, as on-farm hatching does not only include the offering of early feeding but also the absence of transport of day-old chicks and of other potentially stressful hatchery processing procedures.

Therefore, the objectives of the present study were first to test the possibility/success of hatching eggs (d 18) in a special on-farm unit near the broiler-rearing house. Additionally, to the best of our knowledge, it is the first study investigating the possibility of rearing newly hatched chicks with immediate access to feed and water in the same hatching unit one week prior to transferring them to the conventional broiler house. Secondly, the study aimed to investigate the effects of this new strategy on foot pad health as an animal welfare issue as well as litter quality and growth performance.

## 2. Materials and Methods

The broiler chickens in this study were raised under standardized husbandry conditions and subjected to a standard fattening procedure on the Farm for Education and Research Ruthe, University of Veterinary Medicine Hannover, Foundation, Sarstedt, Germany. Since no interventions were carried out on live animals, the study was not considered an animal experiment according to the Animal Protection Act, and thus did not require approval from the respective authority.

### 2.1. Experimental Design

**Figure 1:**
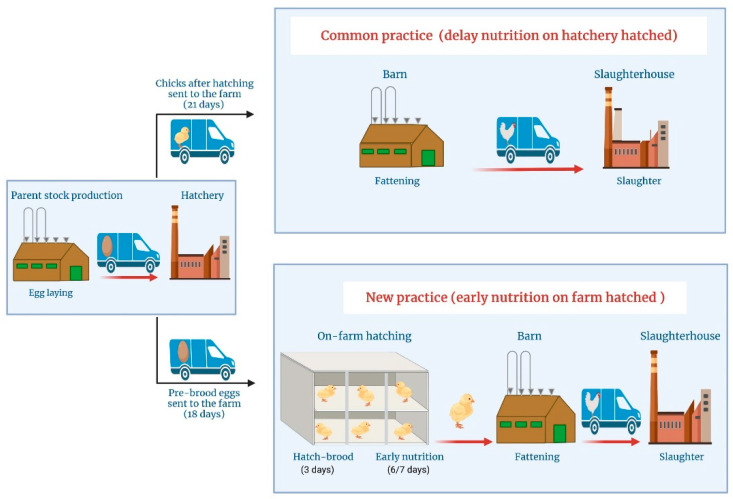
Concept of common hatching practice (delay nutrition on-hatchery hatched) versus the new strategy of early nutrition on-farm hatched. (Figure was created with BioRender.com, in 20 August 2021).

Influences of delayed or early nutrition of broilers were investigated in two subsequent trials upon two-factorial analysis (group “either delay nutrition or early nutrition” and trial repetition “either trial 1 or trial 2”). The concept of common hatching practice (delay nutrition on-hatchery hatched) versus the new strategy of early nutrition on-farm hatched is presented in Figure 1.

In the common hatching practice, the newly hatched chicks were allowed to be transported within the first 72 h after hatching with a maximum transportation period of 24 h without feed and water. In contrast, in the new hatching strategy, the embryonated eggs (d 18) were transported in a special truck and thereafter hatched on-farm in a special hatching unit and reared there for 6/7 days with immediate access to feed and water. Thus, in this new hatchery strategy, there is a combined effect of on-farm hatching and the early access of feed and water. Thereafter, these chicks were transported to the conventional broiler house for rearing in the same facility. Chicks’ ages are expressed as chronological age [16], starting from the end of the hatch window (0 d) until slaughter (33 d).

### 2.2. Housing

The facility consisted of a conventional broiler closed house with a floor pen of 398 m^2^. A wire fence was used in the middle of the stall to separate the control and experimental groups in both trials. The location of groups was swapped between both trials to avoid any effects regarding housing conditions. Generally, each pen was littered with wood shavings to a depth of approximately 1 cm above the floor at the start of the trials. The litter material was covered with chick paper in the stall for the first two days of life to provide access to feed for the chicks. The temperature and humidity in the stables were controlled automatically (Big Dutchman International GmbH, Vechta, Germany) with a regimen of 16L:8D. The light intensity was identical in both groups for the 2 trials. The dark period was between 2100 and 0500 h. Both lights and shutters, which blocked external light, were automatically controlled using timers. The artificial light source in all houses consisted of 2 rows of 14 fluorescent strip lights, running parallel to each other along the length of the house. At the age of two weeks, all chicks received an obligatory Newcastle disease vaccination. The feeders and nipple drinkers (without cups) were installed in the longitudinal pens of the special unit (on-farm hatched). An automatic chain feeding system by the trough and nipple drinkers with drip cups (Big Dutchman International GmbH, Vechta, Germany) were used in the barn. The stocking density was estimated in accordance with the final BW of slaughterhouse data as well as the number of live birds delivered to the slaughterhouse divided by the size of floor pen reared by each group. Stocking densities did not exceed 33 kg/m^2^ at any stage of the production cycle.

### 2.3. Hatching

In comparison to the control group, overlay egg trays (embryonic age: d 18) were obtained from the same commercial hatchery (i.e., eggs had absolutely identical conditions until d 18 of brooding). Thereafter, the eggs were incubated under standard incubation conditions near the conventional broiler house (experimental group: early nutrition on-farm hatched). Three days prior to hatching, the floor temperature was about 34 °C and air temperature was controlled (37.5 °C), while the average humidity was around 60–65% from the housing of the embryonated eggs until hatch based on recommendations of Lourens et al. [31] and Molenaar et al. [32]. The on-farm hatched chicks were reared in six longitudinal pens (1.60 m × 5.00 m) covered with wood shavings. After 6 or 7 days of life on-farm hatched (trials 1 and 2, respectively), the chicks were transferred to the broiler house on the same facility to simulate conventional fattening procedures.

### 2.4. Animals

Two trials were performed with a total of 6900/6850 (trials 1/2) broiler chickens ROSS 308 (BWE-Brüterei Weser-Ems GmbH & Co. KG, Visbek-Rechter-feld, Germany). A total of 3318/3391 chicks (trials 1/2) were reared (after a 3 h transport from the hatchery) in a conventional broiler house (control group; delayed nutrition on-hatchery hatched. In comparison to that, a total of 3582/3459 (trials 1/2, respectively) eggs (embryonic age: d 18) were obtained from the same parent flock of the same commercial hatchery and transported in an air-conditioned van to the research farm facility. The chicks in the control groups (delayed nutrition on-hatchery hatched) did not receive any form of nutrition from hatching till being housed in the conventional broiler house, whereas in the case of the on-farm hatched, the newly hatched chicks had immediate access to water and feed (experimental group: early nutrition on-farm hatched).

### 2.5. Diets and Chemical Analysis

Feed and water were available *ad libitum* for all groups. All birds in both trials were fed commercial pelleted diets throughout the rearing period. The commercial starter diets were offered for the first week of life to all birds. From the beginning of the second week, the birds received a commercial grower diet. The commercial finisher diet was offered from the beginning of the fourth week to all birds. Generally, the commercial diets fed to all birds in both trials were based on wheat grain, yellow corn, soybean meal, and rapeseed meal obtained from MEGA Tierernährung GmbH & Co. KG, Visbek-Rechterfeld, Germany.

The diets were analyzed by the Association of German Agricultural Analytic and Research Institutes (VDLUFA) methods [33]. The dry matter (DM) content was determined mathematically by weighing before and after drying the samples at 103 °C. The muffle furnace was used to detect the crude ash content by weighing the samples before and after combustion at 600 °C. The Dumas incineration method (Vario Max, Elementar, Analysensysteme GmbH, Langenselbold, Germany) was applied to measure the total N content. Moreover, the crude fat content was measured by the Soxhlet apparatus using a standard protocol. The crude fiber content was determined by washing the samples in diluted sulfuric acid and sodium hydroxide (Fibertec^TM^ 2010, Foss Analytical A/S, Hillerød, Denmark). A polarimetric method was used to determine the starch content of the diets (Schmidt + Haensch GmbH & Co., Berlin, Germany). Sugar in the samples was analyzed by using the Luff–Schoorl method, while the atomic absorption spectrometry was used to analyze the minerals (Unicam Solaar 116, Thermo Fisher Scientific GmbH, Dreieich, Germany). Finally, ion-exchange chromatography (AA analyzer LC 3000, Biotronik Wissenschaftliche Geräte GmbH, Maintal, Germany) was used to analyze amino acid contents. The chemical composition of the starter, grower and finisher diets is presented in Table 1.

### 2.6. Excreta and Litter Quality and Foot pad Scoring

Fresh pure excreta samples (*n* = 9) from each group were collected (and pooled) weekly to measure DM content. Additionally, litter samples (*n* = 9) for measuring the DM content were collected (on the same day as the excreta sampling) from nine locations/spots (six peripheral and three central samples within a standardized procedure, i.e., equal distances to feeder and drinker lines and the peripheral wall). The foot pads (only the central plantar) of the birds (50 birds/group) were scored weekly on a scale from 0 to 7 in accordance with Mayne et al. [30]: score 0 = healthy skin, score 7 = more than 50% of foot pad area is necrotic. The average scoring of both legs was performed for each bird. A photograph of the foot pad scoring was shown in a previous study [34]. Foot pad scores were determined by forming the average of the scores for both feet of each animal.

### 2.7. Performance Parameters

In both trials, the individual BW was recorded at d 0, d 4, and d 6/7, and thereafter once a week by catching 50 birds randomly from each group as performed previously by Abd El-Wahab et al. [35]. BW was recorded in the farm by using digital scale (BAT 1, VEIT Electronics, Moravany, the Czech Republic). Moreover, animal losses were recorded in each group. In addition, the final BW data were obtained from the slaughterhouse per group for each trial.

### 2.8. Statistical Analysis

The statistical analysis was performed using the Statistical Analysis System for Windows, the SAS^®^ Enterprise Guide^®^, version 9.3 (SAS Institute Inc., Cary, NC, USA). The BW and foot pad scores were analyzed for the individual animals; further values, i.e., litter and excreta DM contents were analyzed at the group level. For most parameters, mean values, as well as the standard deviation of the mean (SD), were calculated. A test for normal distribution was performed using the Shapiro–Wilk test and normally distributed data were checked for significant differences with the Ryan–Einot–Gabriel–Welsch test (simple Anova). Not normally distributed data were checked for significant differences with a Kruskal–Wallis test followed by a Wilcoxon two-sample test. In order to determine the influence of the two factors (group type and trial repetition) in the first and second trials, the *p*-values were determined using two-factorial ANOVA. Differences with a significant level of *p* < 0.05 were considered significant.

## 3. Results

### 3.1. Dry Matter Content of Excreta

The results of two-factorial analysis regards to excreta DM content in both trials are presented in Table 2. Only at d 6/7 of life did the DM content of excreta show significant differences for the group effect (*p* < 0.05). A significant effect of the trial repetition was noted on the excreta DM content throughout the rearing period except at d 4 of life. However, the significant effect of the group x trial was observed only at d 32 of life.

### 3.2. Litter Quality

The results of litter DM content upon using two-factorial analysis in both trials are presented in Table 3. The data revealed that no significant differences in litter DM content between delayed nutrition on-hatchery hatched (control group) or early nutrition on-farm hatched (experimental group) during the rearing period (except at d 6/7 and d 14 of life). Nevertheless, significant differences in litter DM content were noted regarding the effects of the trial repetition (except at d 0, d 6/7 and d 14 of life). However, group x trial as factors on litter DM content did not show any significant effects throughout the rearing period (except at d 21 and d 28 of life).

### 3.3. FPD Scoring

At the beginning of the trial, the foot pads of all birds were healthy in each trial (Table 4). Interestingly, birds of early nutrition on-farm hatched (experimental group) exhibited significantly lower FPD scores from d 14 until d 32 of life using two-factorial analysis compared to birds of delayed nutrition on-hatchery hatched (control group). The trial repetition had a significant effect on the FPD scoring only at d 4, d 21 and d 32 of life. However, the group × trial showed significant differences in FPD scoring only at d 14, d 28, and d 32 of life.

### 3.4. Performance Data and Mortality Rate

Results for feed and water intakes were monitored for both groups together, as it was difficult to determine these parameters for each group separately. Two-factorial analysis revealed that birds of early nutrition on-farm hatched (experimental group) showed a significantly higher BW during the entire rearing period compared to birds of delayed nutrition on-hatchery hatched (control group) (Table 5). The trial repetition had a significant influence on the BW independent of group (except at d 21 and d 32 of life). However, group × trial affected the BW significantly only at d 0, 4, and 28 of life.

According to the data of the slaughterhouse (Table 6), the final BW (d 33) was higher for the birds of early nutrition on-farm hatched (experimental group) (1893 g and 1911 g for trials 1 and 2, respectively) in comparison to delayed nutrition on-hatchery hatched (control group) (1817 g and 1795 g for trials 1 and 2, respectively). The mortality rate in the first week of life was 1.81% and 1.24%, in trials 1 and 2, respectively, for the group of delayed nutrition on-hatchery hatched (control group) vs. 1.28% and 0.87% for the group of early nutrition on-farm hatched (experimental group) in trials 1 and 2, respectively. From week 2 onwards the mortality rate in the first trial was about 3.79% in the early nutrition on-farm hatched (experimental group) vs. 3.62% for delayed nutrition on-hatchery hatched (control group). Nevertheless, in the second trial, the mortality rate (d 8–d 33) was 5.66% in the early nutrition on-farm hatched (experimental group), while it was 6.75% for the delayed nutrition on-hatchery hatched (control group).

## 4. Discussion

Litter quality is one of the essential factors affecting the welfare of broilers since birds spend all of their lives on litter material. Additionally, litter management is a crucial step for foot pad health. Moreover, FPD is considered an indicator of housing conditions and the welfare of poultry [27]. In the current study, litter quality and/or DM content were almost similar between both groups in trials 1 and 2 (except at d 6/7 and d 14). The main reason for drier litter at d 6/7 and d 14 was that the chicks in the early nutrition on-farm hatched group were transferred from their hatched pens to a new/fresh litter material compared to the delayed nutrition on-hatchery hatched groups. In the present study, gross lesions concerning the foot pad health of the broilers could be determined in both trials. Interestingly, in our study, from d 14 of life up until the end of the fattening period, the early nutrition on-farm hatched group resulted in healthier foot pads than the delayed nutrition on-hatchery hatched groups. It is well known that many different factors could affect FPD. However, wet litter alone may be the cause in most cases [27,30]. Standing on wet litter brings the broilers feet in constant contact with moisture and it has been suggested to induce FPD [36]. Additionally, a reduced prevalence of FPD has also been found in fast-growing broilers hatched on-farm [1,24,25], a painful condition that develops due to poor litter quality [37]. In agreement with this, de Jong et al. [24] found the dry matter content of litter to be higher in the on-farm hatching system. It has to be mentioned that although birds in the experimental group had a higher BW in both trials, i.e., an assumed high feed intake as well as high water intake, broilers still had a very low incidence of FPD. There are two plausible explanations for the low severity of FPD from d 14 of life in this study. Firstly, the possible effect of fresh litter used at d 6/7, resulting in more dry litter remaining dry for a longer period of time before becoming wet via excreta. Thus, providing fresh and/or new for the early nutrition on-farm hatched chicks led to a delay in the onset of incidence and severity of foot pad lesions. Secondly, another explanation could be the indirect effect of the early feeding regime which helped in developing healthy feet.

Early broiler feeding post-hatch is one of the priorities that could affect growth performance, feed efficiency and health [38]. It has to be explained that in our study, it was not possible to explain the effect of feed and water intakes due to both groups being fed from the same automatic feeder and drinker lines in the same barn. Moreover, the day of moving of birds either on d 6 or d 7 (for first and second trials, respectively) in the early nutrition on-farm hatched group was simply to set an upper BW up to which the ventilation, temperature and all other parameters could be kept optimal. This BW was reached in the first trial on day 6 of life, while in the second trial, it was achieved only on day 7 of life. In the current study, it was shown that delayed nutrition on-hatchery hatched chicks without immediate access to feed and water after hatch resulted in impaired growth performance during the entire rearing period. Gonzales et al. [39] observed a reduction in BW of broiler chicks at d 7 when fasting for 30 h post-hatch or longer. Our results also agree with Noy and Sklan [40] who reported a decrease in the BW of chicks kept for 34 h or longer until d 14 compared to chicks offered immediate access to feed and water. Weight loss of an animal in transit has been mainly attributed to dehydration, which has been found to be related to transport duration caused by an increase in evaporative heat loss [41,42]. According to Hollemans et al. [22], the low BW of delayed nutrition chicks until 28 d of age was due to impaired organ development and dehydration during delayed access to nutrition [17,18]. In a review by Noy and Uni [43], it was mentioned that during the hatching process, embryos deplete their glycogen reserves. This leads to the mobilization and metabolism of protein from skeletal muscle for energy. These two factors are thought to be another reason for weight loss due to post-hatch delayed feeding. Therefore, weight loss has to be considered as major welfare and probably early growth performance problems associated with newly hatched chicks in transit to the rearing house [39]. Of importance, in the present study, the first hatched chicks had direct access to feed and water (without having to experience any delayed feeding or stress caused by transportation) and without a need to wait until the end of the hatching window. Early nutrition of chicks can provide readily available energy to assist in restoring hepatic glycogen stores and maintain high body temperature during initial post-embryonic days [26]. Additionally, it is understood that early access to feed is essential for the good immunity and health of chicks [44].

Denying chicks feed and water in our study had adverse effects on mortality. It was observed that the mortality rate was lower in the first week of life in groups of early nutrition on-farm hatched (0.53% and 0.37% in trials 1 and 2, respectively) compared to those of delayed nutrition on-hatchery hatched. Delayed access to feed has been shown to increase mortality in broilers [45]. Total mortality at d 42 was already higher after 48 h post-hatch feed and water deprivation (common in commercial practice) compared to early-fed chickens [13]. Vieira and Moran [46] reported that chicks experiencing a delay in being housed had higher mortality compared to chicks fed immediately after hatching. The delayed housing of chicks has been reported to cause poor response to vaccination, impairment in the gastrointestinal tract and immune system development, poor resistance to diseases and pathogens [47]. This effect was reflected in a tendency for lower mortality in early nutrition on-farm hatched groups in the first week as compared with the delayed nutrition on-hatchery hatched groups. In the present experiment, all groups were housed on a single farm and thus were exposed to similar management practices. Thus, it could be stated that early access to feed (early nutrition) is critical for the good immunity and health of chicks and consequently decreases the mortality rate especially in the early days of life. It cannot be excluded that other factors such as chick handling [14], dust, and pathogens in the hatchery [15] and stress resulting from the transport as day-old chicks [48] in the delayed nutrition on-hatchery hatched groups may also have contributed to the effect on mortality, but that merits further studies as data are scarce. Moreover, in ovo feeding of specific micronutrients to embryonated eggs could enhance the health status of the chicks as was reported recently by Gouda et al. [49].

## 5. Conclusions

Delay nutrition was associated with higher total mortality as well as increased incidence of foot pad lesions. Moreover, the results indicate a negative effect of post-hatch feed and water deprivation (delayed nutrition) on growth performance. Early nutrition can be of critical importance to improve the well-being of birds by reducing mortality and foot pad lesions overall in addition to enhancing nutrient utilization and optimizing growth performance. Thus, providing a source of feed and water as early as possible seems to be beneficial to enhance animal welfare as well as performance.

## Figures and Tables

**Table 1:** Analyzed chemical composition of experimental diets.

Item (g/kg DM) ^2^	Commercial Diets ^1^					
	Starter		Grower		Finisher	
	T1	T2	T1	T2	T1	T2
DM	898	893	891	894	891	891
Crude ash	57.6	57.1	60.9	57.8	55.9	60.3
Crude protein	219	222	213	214.3	222	223
Crude fat	43.8	47.0	50.7	55.3	52.4	51.2
Crude fiber	28.4	28.7	38.3	38.1	30.7	34.1
Starch	490	486	469	462	480	471
Sugar	44.5	46.1	46.6	48.0	48.3	48.0
Metabolizable energy (MJ/kg) ^3^	13.7	13.8	13.5	13.6	13.9	13.7
Calcium	9.40	10.1	7.60	8.31	7.00	8.37
Magnesium	1.80	1.76	1.88	1.86	1.76	1.77
Phosphorus	7.69	7.18	5.89	5.95	5.56	5.19
Sodium	1.58	1.41	1.76	1.45	1.42	1.45
Potassium	9.30	9.35	9.23	9.23	8.86	8.91
Chloride	2.15	1.96	2.20	2.08	2.03	2.02
Copper (mg/kg DM)	26.7	23.3	28.2	23.7	24.7	23.8
Zinc (mg/kg DM)	107.2	94.8	104	108	91.3	101
Iron (mg/kg DM)	267	276	329	298	309	277
Manganese (mg/kg DM)	144	141	137	130	112	134
Arginine	13.5	12.9	12.9	12.6	13.4	12.6
Cysteine	3.71	3.90	3.68	3.35	4.07	3.35
Isoleucine	8.86	8.49	8.44	8.37	8.56	8.37
Leucine	16.8	16.0	15.7	15.7	16.1	15.7
Lysine	13.5	12.8	13.4	12.2	12.6	12.2
Methionine ^4^	6.58	6.46	3.05	2.57	5.97	2.57
Phenylalanine	10.6	10.1	10.0	9.69	10.3	9.69
Threonine	8.25	7.91	7.98	8.54	8.45	8.54
Valine	9.92	9.68	10.1	9.69	10.1	9.69

^1^ T1 = first trial; T2 = second trial; ^2^ DM = dry matter; ^3^ Metabolizable energy (MJ/kg) = 0.01551 × g/kg crude protein + 0.03431 × g/kg crude fat + 0.01669 × g/kg starch + 0.01301 × g/kg sugar; ^4^ Methionine hydroxy analog was added only to grower commercial diets at a percentage of 0.28%.

**Table 2:** Dry matter content (%) of excreta depending on different groups and different trials (*n* = 18 each, except at d 4; *n* = 6) using two-factorial analysis.

Trial	Group	Day of Life					
		4	6/7	14	21	28	32
1 *	Control	21.7 ± 0.404	22.8 ± 0.250	22.8 ± 0.479	23.3 ± 0.580	22.9 ± 0.557	23.1 ± 0.263
	Experimental	22.1 ± 0.404	23.0 ± 0.238	22.8 ± 0.939	23.2 ± 0.359	22.7 ± 0.714	23.2 ± 0.238
2 *	Control	22.2 ± 0.964	20.1 ± 0.668	24.3 ± 0.512	26.8 ± 1.59	29.7 ± 2.80	25.4 ± 0.624
	Experimental	23.3 ± 1.00	21.1 ± 0.374	23.5 ± 0.526	26.5 ± 1.02	27.7 ± 2.11	26.2 ± 1.27
*p*-value	Group	0.122	0.016	0.223	0.697	0.248	0.280
	Trial	0.096	<0.001	0.005	<0.001	<0.001	<0.001
	Group × Trial	0.443	0.073	0.176	0.922	0.327	0.373

* There were no significant differences between groups at each time point within each trial.

**Table 3:** Dry matter content (%) of litter depending on different groups and different trials (*n* = 18 each, except at d 0 and d 4; *n* = 6) using two-factorial analysis.

Trial	Group	Day of Life						
		0	4	6/7	14	21	28	32
1	Control	91.8 ^a^ ± 0.404	87.9 ^a^ ± 1.86	83.7 ^a^ ± 3.11	76.9 ^a^ ± 4.51	69.0 ^b^ ± 2.60	66.1 ^b^ ± 1.80	63.2 ^a^ ± 2.09
	Experimental	91.8 ^a^ ± 0.436	85.2 ^a^ ± 1.82	72.1 ^b^ ± 0.797	80.2 ^a^ ± 1.30	73.6 ^a^ ± 0.601	69.6 ^a^ ± 1.03	65.4 ^a^ ± 1.68
2	Control	91.9 ^a^ ± 0.361	82.3 ^a^ ± 6.83	83.8 ^a^ ± 5.36	74.6 ^a^ ± 3.04	77.4 ^a^ ± 3.61	80.3 ^a^ ± 1.97	73.2 ^a^ ± 3.66
	Experimental	92.0 ^a^ ± 0.252	75.2 ^a^ ± 3.33	72.0 ^b^ ± 10.8	77.9 ^a^ ± 1.86	74.3 ^a^ ± 2.57	78.5 ^a^ ± 2.26	74.4 ^a^ ± 2.12
*p* -value	Group	0.706	0.066	<0.001	0.013	0.490	0.305	0.122
	Trial	0.415	0.010	0.992	0.064	<0.001	<0.001	<0.001
	Group × Trial	0.821	0.377	0.972	0.995	0.002	0.002	0.642

^a,b^ Means in a column within each trial with different superscripts differ significantly (*p* < 0.05).

**Table 4:** FPD scoring of broilers depending on different diets and different trials (*n* = 100 each) using two-factorial analysis.

Trial	Group	Day of Life					
		4	6/7	14	21	28	32
1	Control	0.020 ^a^ ± 0.141	0.410 ^a^ ± 0.668	1.77 ^a^ ± 1.71	2.04 ^a^ ± 2.04	1.78 ^a^ ± 1.93	2.54 ^a^ ± 2.30
	Experimental	0.120 ^a^ ± 0.372	0.360 ^a^ ± 0.452	0.870 ^b^ ± 0.781	1.02 ^b^ ± 0.742	0.910 ^b^ ± 0.793	1.18 ^b^ ± 1.20
2	Control	0.180 ^a^ ± 0.414	0.590 ^a^ ± 0.560	1.04 ^a^ ± 0.807	1.31 ^a^ ± 0.931	1.22 ^a^ ± 0.648	1.29 ^a^ ± 0.840
	Experimental	0.240 ^a^ ± 0.381	0.390 ^a^ ± 0.455	1.02 ^a^ ± 0.827	0.920 ^b^ ± 0.547	1.01 ^b^ ± 0.237	1.09 ^b^ ± 0.241
*p*-value	Group	0.102	0.104	0.004	<0.001	<0.001	<0.001
	Trial	0.005	0.172	0.064	0.016	0.140	0.001
	Group × Trial	0.682	0.328	0.005	0.067	0.035	0.003

^a,b^ Means in a column within each trial with different superscripts differ significantly (*p* < 0.05).

**Table 5:** Average BW (g) depending on different diets and different trials (*n* = 100 each) using two-factorial analysis.

Trial	Group	Day of Life						
		0	4	6/7	14	21	28	32
1	Control	46.2 ^a^ ± 3.32	111 ^a^ ± 9.29	167 ^a^ ± 14.8	475 ^b^ ± 66.8	902 ^b^ ± 101	1524 ^a^ ± 181	1760 ^b^ ± 204
	Experimental	47.6 ^a^ ± 3.67	113 ^a^ ± 9.38	169 ^a^ ± 13.0	509 ^a^ ± 60.8	954 ^a^ ± 109	1575 ^a^ ± 184	1911 ^a^ ± 223
2	Control	40.3 ^b^ ± 3.29	96.2 ^b^ ± 8.21	160 ^b^ ± 13.4	419 ^b^ ± 50.4	885 ^b^ ± 126	1381 ^b^ ± 191	1736 ^b^ ± 185
	Experimental	47.5 ^a^ ± 2.75	104 ^a^ ± 9.39	167 ^a^ ± 14.3	462 ^a^ ± 51.3	966 ^a^ ± 76.7	1541 ^a^ ± 142	1883 ^a^ ± 177
*p*-value	Group	<0.001	<0.001	0.025	<0.001	<0.001	<0.001	<0.001
	Trial	<0.001	<0.001	0.024	<0.001	0.847	0.001	0.349
	Group × Trial	<0.001	0.017	0.230	0.551	0.341	0.029	0.935

^a,b^ Means in a column within each trial with different superscripts differ significantly (*p* < 0.05).

**Table 6:** Overall farm and slaughterhouse data for both groups for trials 1 and 2 during the entire experimental period (d 0–d 33).

Trial	Parameter	Control Group	Experimental Group
1	Total live birds at d 0 (*n*)	3318	3582
	Total live birds at d 6 (*n*)	3258	3536
	Total live birds at d 33 (*n*)	3140	3402
	Mortality rate from d 0–d 6 (%)	1.81	1.28
	Mortality rate from d 7–d 33 (%)	3.62	3.79
	BW at 0 d (g)	46	48
	BW at 33 d (g), slaughterhouse data	1817	1893
	BWG from d 0–d 33 (g)	1771	1845
	Stocking density (kg/m^2^) ^1^	28.7	32.4
2	Total live birds at d 0 (*n*)	3391	3459
	Total live birds at d 7 (*n*)	3349	3429
	Total live birds at d 33 (*n*)	3092	3196
	Mortality rate from d 0–d 7 (%)	1.24	0.87
	Mortality rate from d 8–d 33 (%)	6.75	5.66
	BW at 0 d (g)	40	48
	BW at 33 d (g), slaughterhouse data	1795	1911
	BWG from d 0–d 33 (g)	1755	1863
	Stocking density (kg/m^2^) ^1^	27.9	30.7

^1^ The facility consisted of a conventional broiler house with a floor pen/group of 199 m^2^.

## Data Availability

The data presented in this study are available in this manuscript.
